# Thoughts about fertility among female adolescents and young adults with cancer: a qualitative study

**DOI:** 10.1007/s00520-023-07887-0

**Published:** 2023-06-26

**Authors:** Line Bentsen, Helle Pappot, Maiken Hjerming, Signe Hanghøj

**Affiliations:** 1 Department of Oncology, Copenhagen University Hospital, Rigshospitalet, Blegdamsvej 9, 2100 Copenhagen, Denmark; 2 Department of Hematology, Copenhagen University Hospital, Rigshospitalet, Blegdamsvej 9, 2100 Copenhagen, Denmark

**Keywords:** Adolescents and young adults, Cancer, Infertility, Concerns, Family formation, Fertility preservation

## Abstract

**Purpose:**

Nine hundred female adolescents and young adults (AYAs) aged 15–39 are diagnosed with cancer in Denmark annually. Advances in cancer therapy have led to increased long-term survival; however, a serious side effect of cancer therapy is reduced fertility. The aim of our study was to explore the thoughts about fertility among female AYAs with cancer.

**Methods:**

Our study was conducted from September 2020 to March 2021 at the Copenhagen University Hospital, Rigshospitalet. Inclusion criteria were female AYAs with cancer aged 18–39. Twelve individual, semi-structured, qualitative interviews were performed with female AYAs with cancer (20–35 years). Data were analysed using thematic analysis.

**Results:**

Four main themes were found: (1) the female AYAs held on to a hope of having children in the future; (2) the female AYAs experienced time pressure and waiting time as a sprint as well as a marathon; (3) the female AYAs faced existential and ethical choices about survival and family formation; and (4) the female AYAs felt a loss of control of their bodies.

**Conclusion:**

Our study contributes with knowledge on how important holding on to the hope of children in the future is among female AYAs with cancer. Meanwhile, they are frustrated by the rushed decision on fertility preservation at diagnosis. The female AYAs also have existential and ethical concerns related to the choice of cancer therapy and fertility preservation. Finally, they suffer from altered body image, loss of femininity, and body control due to hormone therapy.

## Introduction

In Denmark, approximately 900 female adolescents and young adults (AYAs) aged 15–39 are diagnosed with cancer every year [1]. For this patient group, identity development and reproductive health are essential themes given their current life situation [2]. This does not necessarily change with a cancer diagnosis — on the contrary, the issues may be reinforced, both at onset of a cancer diagnosis and into survivorship [3, 4].

Cancer therapy is often associated with permanent reduced fertility or even infertility [5]. For this reason, several international guidelines on fertility preservation in AYAs with cancer have recently been presented [6–9]. Despite these, numerous studies indicate a continuing problem regarding inadequate oncofertility counselling to AYAs with cancer [10]. We have previously explored and found that young female AYAs with cancer experienced the oncofertility counselling as unsystematized. The topic was often initiated by the patient and the information given varied, leading to mistrust and frustration [11]. Also, a minority of AYAs with cancer are referred to fertility specialists for counselling and fertility preservation prior to initiation of cancer therapy [12]. Compared to males, females are less likely to be referred and to receive fertility-preserving treatment [13].

Female AYAs often have fertility concerns when diagnosed with cancer, during their cancer course and into survivorship [14–16]. Biological motherhood can be unwillingly postponed due to recommendations on not becoming pregnant in fear of teratogenic risk and the risk of recurrence. For young breast cancer patients undergoing adjuvant endocrine therapy, this may be up to 5–10 years [17]. This makes young cancer patients vulnerable to the risk of non-adherence to their cancer treatment [18, 19].

To improve the handling of AYAs with cancer and their fertility challenges, more knowledge is needed on this topic from the patients’ viewpoint. We believe it is important for health professionals to have qualitative in-depth knowledge of the thoughts and beliefs held by AYAs related to fertility to enable appropriate oncofertility counselling. The purpose of this qualitative study was to explore thoughts about fertility among female AYAs with cancer.

## Materials and methods

### Setting

From September 2020 to March 2021, the study was conducted at the youth support centre and social organization, Kræftværket, which helps and supports AYAs with cancer at the Copenhagen University Hospital, Rigshospitalet.

### Recruitment and participants

RN and Kræftværket youth coordinator MJ informed Kræftværket users about the research project, including the researchers, prior to recruitment. Inclusion criteria were female AYAs with cancer (18–39 years). Participants were recruited through Kræftværket, by way of a written invitation posted on a closed Facebook group with approximately 320 members (consisting of AYAs with cancer or survivors).

#### Data collection

The semi-structured interview guide included questions regarding expectations about having children in the future and thoughts related to this in with the context of their cancer diagnosis and therapy. The guide also addressed whether they had received oncofertility counselling and/or fertility tests and/or fertility preservation. See Table 1. The interviews were conducted at Kræftværket or in the Department of Oncology, Copenhagen University Hospital, Rigshospitalet. Two participants brought next of kin to the interview. LB audio recorded the interviews electronically and transcribed the interviews verbatim into Danish. A professional translator subsequently translated the quotations into English.

**Table 1:** Sample questions from the semi-structured interview guide

What were your plans/wishes for family formation prior to the cancer diagnosis? — Have they changed since the cancer diagnosis? What were your thoughts on family planning/having more children prior to the cancer diagnosis? Describe your expectations, prior to the cancer diagnosis, of the possibility of becoming pregnant What are your thoughts now regarding the possibility of having children in the future? What were your thoughts about fertility at diagnosis and during your time with cancer? Describe the fertility counselling you received at diagnosis and during cancer treatment Did you have your fertility tested prior to/during your time with cancer? Have you had fertility preservation? Have you been in fertility treatment since you have become clear of cancer?	

### Analysis

The analysis of this study was inspired by a thematic analysis approach rooted in Malterud’s systematic text condensation [20] and Braun and Clark’s reflexive thematic analysis [21]. The study was based on a phenomenological approach, and data was analysed inductively, based on coding and themes that arose from the content in the data. The following steps were conducted: (1) the recorded interviews were read several times by LB and SH, while noting possible codes; (2) relevant codes for the research question were discussed between LB and SH after several readings; (3) preliminary themes were developed by LB and SH on the basis of the codes; (4) SH determined the content of each preliminary theme and chose a title for each theme; (5) the research group discussed the themes and allocation of codes until agreement on the final themes was reached; and, finally, (6) SH wrote the final content for the themes, while LB prepared and wrote the article, based on the analysis.

In total, twelve participants were interviewed. Based on the findings of preliminary themes in the ten first interviews, data saturation [22] was discussed and accepted in step three of the six-step analysis method in the study. Two more participants were interviewed where no additional themes were found.

To validate the design, the interview guide ensured that the participants were asked the same number and variety of questions. Rigor and reliability were ensured using the abovementioned six-step analysis, with repeated readings of the transcripts. Furthermore, themes were found independently by the first and last author, respectively, and all themes were discussed in the author group to ensure consensus.

Prior to this study, LB underwent research training in qualitative research guided by senior researcher SH, who has previously conducted several qualitative studies using the above-mentioned method.

### Ethics

Approval of the project was given on 1 September 2020 by the national Data Protection Agency in the Capital Region (P-2020-849). Verbal and written information regarding the aim and study design was given to the participants, including information on anonymity and the opportunity to withdraw their consent at any time without treatment consequences. Written informed consent was obtained from all participants prior to the interviews. Pursuant to Danish law, this study is excepted from ethical approval.

## Results

Twelve female AYAs who were eligible as study participants agreed to participate. Therefore, 12 individual, qualitative, semi-structured interviews were conducted, lasting on average 33 min (between 23 and 59 min). The age interval in the study was 20–35 (mean age 28). See Table 2. From the interviews, four themes were identified. See Table 3.

**Table 2:** Demographic and clinical variable

	Participants *n*=12
Mean age (range)	28 (20–35 years)
Ongoing cancer therapy	4
Ongoing follow-up	8
Relationship status	
In a relationship	8
Single	4
Education	
High school	4
Bachelor’s degree	2
Master’s degree	6
Cancer type	
Acute myeloid leukaemia	1
Acute lymphoblastic leukaemia	1
Brain cancer	1
Breast cancer	2
Hodgkin’s lymphoma	3
Non-Hodgkin’s lymphoma	1
Ovarian cancer	3
Fertility preservation	
Oocyte/ovarian cryopreservation	3
+ Goserelin	1
Only Goserelin	2
No fertility preservation	7

**Table 3:** Themes and subthemes

Theme	Summary of the themes
*Hope:* The female AYAs held on to a strong hope of having children in the future	Expectations of children in the future vanished Hope of future children was fueled by fertility preservation and the egg reserve Hope strengthened the feeling of normality and identity
*Time:* The female AYAs experienced time pressure and waiting time as a sprint and a marathon	Quick clarification of the wish for children was necessary A race against time to initiate a fertility-preserving treatment Long waiting time for normal cycle and green light from the doctors
*Choice:* The female AYAs faced existential and ethical choices about survival and family formation	Choice of cancer therapy influenced long-term survival and fertility Doctors prioritized survival over fertility Ethical dilemma regarding oocytes or embryos
*Body:* The female AYAs lost control of their bodies	The body was physically separated from its fertility The body was controlled by medicine and doctors A necessity to take control of the medicine’s half-life in the body

### Theme 1 “Hope”

#### The female AYAs held on to a strong hope of having children in the future

For several of the participants, fertility did not preoccupy them prior to their cancer diagnosis. They had optimistic expectations of being able to conceive when the time was right. However, some of the participants had a prior concern if they were diagnosed with polycystic ovary syndrome or if their mothers had had difficulty becoming pregnant. Most participants’ expectation of having children in the future changed when diagnosed with cancer because their fertility was in risk of being seriously reduced due to cancer therapy. Thoughts about reduced fertility became overwhelming and frightening for several of the participants. They were anxious and insecure about not knowing their fertility status after cancer therapy. However, they held on to a hope of having children in the future (Table 4: Quote 1).

**Table 4:** Themes, subthemes, and quotes. Each subtheme is represented by at least one quote in the table

Themes	Subthemes	Quotes
Hope	Expectations of children in the future vanished	*Quote 1:* “*I thought I could easily become pregnant. [After the treatment] I just think I have stuck to the hope that it can hopefully happen” (Interview 9)*
	Hope of future children was fuelled by fertility preservation and the egg reserve	*Quote 2:* “*I asked a lot about the possibilities if I had an ovary removed. I was told that it leaves a 30% chance that one can have a child afterwards” (interview 2)* *Quote 3: “They (the doctors) said that I would be able to have children afterwards. So of course it was nice to be told that, but I just still think I was, like, are you sure now? A lot like that, because afterwards it’s too late, now that I have not had any oocytes taken out or anything […] They measured how many eggs I had left, where I then also got the feeling that it can also change, […] it would also be a bit annoying, like, if all of a sudden they [the oocytes] were gone” (interview 3)*
	Hope strengthened the feeling of normality and identity	*Quote 4:* “*I have received 30 radiation treatments, they have not helped, I have received chemo pills, they have not helped […] and despite all that, when I look to my future, I see myself being married and having children […] it is the dream, and you can say, dreams are also allowed to come true*” *(Interview 4)*
Time	Quick clarification of the wish for children was necessary	*Quote 5:* “*In such a situation, you find out if you actually want to have children, that is, if it is something you have a desire for. I found out fairly quickly*” *(interview 12)*
	A race against time to initiate a fertility-preserving treatment	*Quote 6:* “*So the process is like, I get diagnosed with cancer on Monday, on Wednesday we talk to the doctors about all this regarding fertility preservation taking an ovary out. The following Monday I will have surgery, I will have my cancer removed. Tuesday morning I sit and inject myself with hormones*” *(Interview 11)*
	Long waiting time for normal cycle and green light from the doctors	*Quote 7:* “*Then, we also knew all of a sudden that it would take 2.5 years before we would be finished, and then it would take an extra year, and he is just a few years older than me […] so it can definitely be difficult*” *(Interview 8)* *Quote 8:* “*How do you know as a 20-year-old when it is time to have children, because then came all those planning things, so that you got almost stress in the body over having to plan like that, what the next 10 years would look like*” *(Interview 6)*
Choice	Choice of cancer therapy influenced long-term survival and fertility	*Quote 9: “I chose a longer cancer treatment with poorer chances of long-term survival and increased risk of relapse in hope of some remained fertility – even though I was not guaranteed this. I have been more terrified of surviving all the chemo and all the cancer and then losing my fertility, than not surviving at all*” *(Interview 2)*
	Doctors prioritized survival over fertility	*Quote 10:* “*I just feel like the doctor, when you have cancer, it’s like the doctors kind of take everything and throw it away, so for them, nothing is more important than making one healthy. And that’s okay too. But the problem is also just that they forget a little that we also have a life afterwards” (Interview 4)*
	Ethical dilemma regarding oocytes or embryos	*Quote 11*: *“The thing about having oocytes taken out is that you have to decide whether those eggs should be fertilized or not. Which was a huge decision because, I’m pretty sure we need to fertilize the eggs, because that’s what the doctors suggest, that it clearly provides the greatest opportunity to have a child afterwards, so I think, that’s what we’ll do. [Boyfriend’s name] doesn’t think the same, because he’s a little scared that, well then all my eggs are in his basket. Because what the doctors then also inform us about is that if [boyfriend’s name] and I split up, then those eggs are destroyed” (Interview 11)*
Body	The body was physically separated from its fertility	*Quote 12: “Actually, where is it now, that my ovary is? Is it frozen somewhere? It’s lying there in a freezer ... I did not know where it was or who I should get hold of, if I wanted to use it, and if I move back to [country] in 10 or 20 years – then I have to take my ovary with me*” *(Interview 1)*
	The body was controlled by medicine and doctors	*Quote 13: “I could not understand why I had so much pain in my back and my joints, and those hot flashes that just kept going, and it also took longer, I know that it is said that menstruation comes back, you expect it, but it took me at least 8 months after” (Interview 7)*
	A necessity to take control of the medicine’s half-life in the body	*Quote 14:* “*I looked at the half-life of this drug, so I actually also controlled when I took these Zoladex, so that the half-life peaked in relation to that, that the concentration peaked inside me in relation to when the different chemo treatments were. Because, as a patient, you are actually told that we (the doctors) have control of your chemo, and your blood tests, we have control of everything, but that Zoladex, you just have to keep track of it yourself” (Interview 11)*

The participants’ hope was often related to fertility preservation. The doctors occasionally stated the chances of fertility in percentages or loose estimates, e.g. “30 % chance” or “fifty-fifty chance”. Some participants linked their hope to the doctor’s estimates (Table 4: Quote 2).

The doctors also instilled hope in the participants after cancer therapy when informing about their oocytes reserve — if there were oocytes, there was hope. However, the participants became uncertain about whether the oocytes reserve changed during treatment and whether the quality of the oocytes decreased after cancer therapy (Table 4: Quote 3).

Many participants hoped that their fertility would be preserved after completion of therapy, despite no fertility preservation treatment and an uncertain prognosis (Table 4: Quote 4). Thus, hoping to become a mother in the future helped to create the participants’ sense of normality and identity.

### Theme 2 “Time”

#### The female AYAs experienced time pressure and waiting time as a sprint and a marathon

Several of the participants had not thought much about their wishes of future family formation prior to their cancer diagnosis. However, suddenly, this concern became very relevant when the participants were diagnosed with cancer (Table 4: Quote 5).

Several of the participants experienced that the decision as to whether to embark on the fertility preservation treatment was under a time pressure. One of the participants described it as a “race against time” to have an ovary operated on, because the cancer therapy had start immediately (Table 4: Quote 6).

Regardless of whether the participants had received fertility-preserving treatment or not, several of the participants also described a difficult waiting time during the long-lasting chemotherapy treatment course, the return of a normal menstrual cycle and the doctor’s approval before trying to become pregnant. So, while the clarification of wishes for children in the future and initiation of fertility-preserving treatment may felt like a sprint, the subsequent waiting time could be compared to a marathon. Not until the waiting time was over, the participants found out the consequences of the cancer treatment. There were no guarantees that it would be possible to conceive (Table 4: Quote 7).

Due to the long waiting time before being allowed to try to become pregnant, the participants experienced a second round of stressful time pressure. Time pressure was experienced in both the youngest and the oldest participants. They all knew that fertility naturally decreases with age, and it was an added stressor that cancer therapy further reduced the chances of pregnancy. Thus, some of the participants in their 30s felt extra pressure to have children before it was too late. The youngest participants however also experienced time pressure. Several of them had not even thought about when they wanted to have children, and suddenly, they felt they had to plan having children in the future much earlier than expected (Table 4: Quote 8).

### Theme 3 “Choice”

#### The female AYAs faced existential and ethical choices about survival and family formation

For several of the participants the recommended cancer therapy would leave them infertile. Therefore, they experienced having to face existential and ethical choices if they considered choosing the next-best cancer treatment. This would possibly leave them with some fertility, but also with a slightly decreased chance of long-term survival compared to the recommended treatment. Some of the participants explained that their concerns about not being able to have children in the future occupied their minds much more than the risk of decreased long-term survival (Table 4: Quote 9).

Furthermore, several participants reported feeling like they were being dictated by the doctors rather than being recommended a cancer treatment. They described that the doctors made treatment choices based on giving them the best chances of survival, as for them it was natural to prioritize survival over fertility. Their hope for children in the future diminished when the doctors only thought about healing. For these participants, it became an existential question about the meaning of the life they were to live after cancer treatment (Table 4: Quote 10). In contrast, one participant took a different attitude as the most important thing for her was long-term survival. She believed that if she did not survive, thinking about having children in the future did not matter at all.

For the participants who underwent fertility preservation, they had to choose between cryopreservation of either an ovary or oocytes. The choice had to be made immediately after diagnosis and could be very difficult and stressful. One of the participants explained that she and her partner faced several difficult dilemmas. First, whether they wanted fertility preservation and then — if they chose to have oocytes taken out — whether they should be fertilized with the partner’s semen or not. If they are subsequently separated from their partner, Danish legislation prohibits the woman in using the fertilized oocytes. It therefore turned out to be an ethically difficult dilemma in relation to the partner’s role and responsibility (Table 4: Quote 11).

### Theme 4 “Body”

#### The female AYAs lost control of their bodies

The uncertainty of their fertility made several of the participants feel that they had an imperfect body. They felt less feminine, and their self-perception and body-image changed. A visible sign of the body’s imperfection was the lack of menstruation, a monthly recurring reminder that the body was not functioning properly. One described herself as a “half human” without her fertility. A general feeling was that fertility had been removed from the body along with the removed ovary. The removed ovary became tangible proof that the fertility was in a freezer and waiting for the body to become healthy enough for it to be put back in. The ovary was so physically far away from the body that one of the participants expressed concern about where it was at all. She did not know how to get hold of it and whether she could transport it back to her home country (Table 4: Quote 12).

Many felt affected by hormonal changes as part of the fertility-preserving treatment. The participants’ young bodies suddenly entered early menopause and felt out of control. Several of the participants were given Goserelin to inhibit the body’s production of sex hormones, which was described as harsh on the body (Table 4: Quote 13).

After the cancer treatment, some participants were treated with oestrogen to restore their menstrual cycle. Once the body had returned to normal after the cancer trajectory, several of the participants were waiting for approval from the doctors to start trying to conceive. Therefore, they experienced that the doctors also controlled their bodies. In contrast, one of the participants tried to take control of her fertility treatment, because she did not feel that the doctors took sufficient responsibility. By managing this herself, some of the lost control of the body was regained. She felt compelled to calculate the half-life of the medicine in the body, to take it at the right time (Table 4: Quote 14).

## Discussion

This explorative qualitative study investigated thoughts about fertility among female AYAs with cancer. The results pointed to four main findings: (1) the female AYAs held on to a hope of having children in the future; (2) the female AYAs experienced time pressure and waiting time as a sprint as well as a marathon; (3) the female AYAs faced existential and ethical choices about survival and family formation; and (4) the female AYAs felt a loss of control of their bodies.

The female AYAs thoughts were characterized by uncertainty and concerns, which is in line with existing literature [14–16, 23]. However, our study contributes with new knowledge on how important holding on to the hope of children in the future is among female AYAs with cancer.

We found that hope of preserved fertility became an important driving force through the cancer trajectory, and it supported the female AYAs’ self-image of normality and identity. In alignment with Yee et al. our results show that, for some participants, their fertility concerns override all other concerns and hope becomes a part of their coping strategy [4]. Others argue that, when fertility is at risk, fertility preservation sustains the hope of fertility and future family formation [24–27]. This is supported by our findings, where fertility preservation prior to cancer therapy increased the AYAs’ hope of maintaining their fertility. However, even AYAs who did not undergo fertility preservation maintained hope of remained fertility. Therefore, healthcare professionals have to make important ethical considerations on how hope is communicated and the role hope plays in the interactions between patients and healthcare professionals in decision-making [28].

In our study, the female AYAs experienced that questions about their wishes for future family formation became very present at diagnosis. Several were concerned about making prompt decision and initiation of fertility-preserving treatment. These findings support previous research, in which the decision about fertility preservation is described as being “under the gun” [29, 30]. We also found that the long waiting time before trying to become pregnant led to a second round of stressful time pressure. This is supported by Gonçalves et al. who describes that the waiting time of postponed desired motherhood, including excessive uncertainty, can cause emotional maladjustments [25]. Despite most of the participants were in a romantic relationship during their cancer trajectory, most women do not mention relationship themes in relation to fertility, suggesting that the question of fertility may relate to the female identity rather than to a parenthood. However, this could be due to the overall purpose of this study exploring the women’s thoughts about fertility during a cancer trajectory rather than the thoughts of young couples affected by cancer in relation to fertility and family planning.

Our results showed that the female AYAs faced existential and ethical dilemmas regarding cancer their therapy fertility. This is aligned with Partridge et al. who found that infertility concerns influenced the cancer treatment decisions for 29% of female AYAs with breast cancer [33]. Also Sobota et al. argues that AYAs with cancer experience a complex process of balancing the wish to survive with the desire to preserve fertility [29].

We also found that the existential and ethical choices were more difficult if the health care providers had an exclusive focus on survival. This echoes previous findings [30, 34]. Both Peate et al. and Gould et al. found that younger patients with cancer preferred an active role in decision-making, but also wished to consider medical advice [35, 36]. This highlights that young female AYAs with cancer call for collaboration with their physicians regarding decision-making about fertility preservation and cancer treatment. In contrast to this, Parton et al. state that decision-making in regard to fertility preservation can also be considered a burden to young female AYAs who are already vulnerable due to their cancer diagnosis [25], arguing for the need of proper oncofertility counselling with a focus on the individual young female cancer patient and her given life situation.

Our results demonstrated difficult ethical considerations about the cryopreservation of either oocytes or embryos because of the difference in the “ownership” in case of separation from or withdrawal of consent of the partner [37]. Rodrigues-Wallberg et al. found that more than half of female AYAs with a partner chose either not to fertilize the oocytes or only fertilized half of the oocytes to ensuring reproductive autonomy in the future [38]. However, an important precondition of making this decision is knowledge of the regulatory framework, which speaks to the need for adequate oncofertility counselling.

We also found that the female AYAs’ body image and self-perception of being feminine changed. They felt that they had an imperfect body and less in control over their bodies. This is also shown by Chow et al. where half of the female AYAs who had gynaecological cancer felt that they had lost their femininity and had been reduced to imperfect or incomplete women [39]. In addition to this, Silva et al. found that the loss of a female reproductive organ had a huge impact on the women’s social identity and functionality [40]. In contrast to this, Paton et al. describes that fertility preservation made the female AYAs feel more in control of their bodies and allowed them to be proactive about their fertility [25].

Our results showed that some of the female AYAs experienced that hormonal medication controlled their bodies and they were affected by hormonal changes. Thus, there is a discrepancy between natural, young, and healthy bodies that function on their own and diseased bodies that are kept going artificially and controlled by medication, because they are out of order. This supports Snöbohn et al. where physical and mental changes in young adults with cancer affected their body image, their rehabilitation and coping capacity [41]. Also Moore et al. found that altered body image among young cancer patients could result in isolation due to negative reactions that re-enforced self-consciousness and potentially affecting interpersonal relationships with family, friends, and intimate partners [42]. This emphasizes the need of proper oncofertility follow-up during the cancer course and into survivorship.

This discussion could further emphasize the central theme that seems to link the four themes identified — uncertainty. The uncertainty that arises with the risk of infertility then spreads to many aspects of these young women’s life: identity (as a woman), trust in doctors, in themselves, and their bodies, in their ability to make plans. Uncertainty is not easily cured, but it can lead to reflection on appropriate care. As other studies also describe, it highlights the paradox and ambivalence described in the four selected themes and is a major theme in explaining the psychosocial needs of these young women, especially during the survivorship period [15, 32]. Even when cancer and fertility treatments are successful, this remains a significant challenge.

### Strengths and limitations

The strength in our study is the use of qualitative interviews, which enabled nuanced descriptions of thoughts of female AYAs with cancer about fertility. The participants represent breadth in demographics including age distribution and relationship status. A possible limitation is that the interviews were all with young women who had a desire for having children in the future, which presents a possible selection bias. Further, the interviews were conducted at a single centre, which limits the possibility of national generalization on this subject.

## Conclusion

Our study contributes with knowledge on how important holding on to the hope of children in the future is among female AYAs with cancer. Meanwhile, they are frustrated by the rushed decision on fertility preservation at diagnosis. The female AYAs also have existential and ethical concerns related to the choice of cancer therapy and fertility preservation. Finally, they suffer from altered body image, loss of femininity and body control due to hormone therapy.
